# The Eyesight of Our School Children

**Published:** 1899-04

**Authors:** 


### The Eyesight of Our School Children.

It is asserted by Dr. B. F. Rogers, in a paper published in the Buffalo *Medical Journal* (March), that our schools are injuring the eyesight of their pupils in a very large number of cases. He says:

"It has been recognized for years that the requirements of school-life result in injury to the eyes of many children. In a number of cities some attention has been paid to the correction of errors of refraction of school children, yet we have not a well-defined and systematically applied plan of preventive measures existing in our schools. The correction of errors of refraction by the correct fitting of glasses to the eyes of such pupils as have trouble already existing should be one of the first measures to receive public attention. But you will agree with me that the ounce of prevention is to be applied by careful attention to the conditions surrounding the smaller pupils in the school-room. Here, in a multitude of cases, may be prevented the development of errors calling for glasses or complete debarment from study. From school reports of different cities I can not find that a very great amount of work has been done in the city schools, except in a general way. In Germany the question has been studied in its most scientific bearings and the practical results applied in a systematic manner."

After quoting various authorities to bear him out in his assertions, Dr. Rogers continues:

"Some of our old school-rooms . . . are nothing more nor less than hotbeds for eye troubles. . . . The principal abnormal conditions which we meet in the eyes of school children are the short-eye or hypermetropia, hypermetropia with astigmatism, and the long-eye or myopia. . . . Myopia is called a disease, which can not be cured and often can not be arrested. Progressive myopia is in every case ominous of evil for the future, for if it continues the eye soon becomes less and less equal to its work. . . . The most dangerous to the eye are the four higher grades in our grammar schools, leading up to the high schools. It is there that myopia commences and increases. There you will find myopes as you ascend the grades—32.5 per cent. of the pupils wearing glasses in the high schools are myopes."

Dr. Cohn, a German authority, found that the number of nearsighted or myopic pupils increased in every school from grade to grade, and he attributed this to improperly adjusted desks. This progressive increase, Dr. Rogers believes, from his own observations in Buffalo, is the case in the United States also. He especially condemns the carelessness with which parents allow their children to begin study without ever testing their vision, so that a slight defect is often allowed by neglect to grow into a great one. Says the doctor:

"The eyes of all children should be tested before admission as pupils to the school. If the vision should prove to be much below the normal, the parents should be advised as to this condition and what should be done. Admission should not be granted until the pupil has sought professional counsel and presents a certificate to that effect. The same rule should apply to children with inflamed eyes, who should not be allowed in the school until a physician's certificate of the non-infectious nature of the disease has been presented. The common wash-basin, towel, and comb should be abandoned entirely."

The conditions that should obtain in schools that the pupil's sight may be kept in the best condition are thus summed up by Dr. Rogers:

"*Light.*—This commences, of course, with location of school lot, its surroundings, the number and location of windows. Quantity and quality of light are modified by the color of walls and shades to the windows. Shades should be hung on the adjustible shade fixtures.

"*Tints.*—Blue, gray, or neutral tints are best for walls.

"*Desks.*—Adjustible desks should be used and placed so that the light falling from the upper sash, when possible, will strike the desk over the pupil's left shoulder.

"*Artificial Light.*—Artificial light is always a bad light for young eyes; school children with myopia or any form of eye-strain should not work or study by artificial light.

"*Writing on Blackboards.*—The writing should be large and legible; if required to be read at fifteen feet, should be large enough to be read at thirty feet.

"*Excessive Work.*—School hours should be carefully adjusted to the strength of the pupil. There should be frequent intervals during school hours for relaxation of the eyes.

"*Length of School Year.*—There is no time gained for the pupil by school sessions the last half of the month of June and first half of September, the two most beautiful months of the year for outdoor recreation."—*Literary Digest.*

